# Abscisic acid induces a transient shift in signaling that enhances NF-κB-mediated parasite killing in the midgut of *Anopheles stephensi* without reducing lifespan or fecundity

**DOI:** 10.1186/s13071-017-2276-4

**Published:** 2017-07-13

**Authors:** Elizabeth K. K. Glennon, Brandi K. Torrevillas, Shannon F. Morrissey, Jadrian M. Ejercito, Shirley Luckhart

**Affiliations:** 1 Department of Medical Microbiology and Immunology, University of California at Davis, Davis, CA USA; 2 Center for Infectious Disease Research, Seattle, WA USA; 3 Department of Entomology, Plant Pathology and Nematology, University of Idaho, Moscow, ID USA; 4 Department of Biological Sciences, University of Idaho, Moscow, ID USA; 5 Department of Entomology, University of California at Riverside, Riverside, CA USA

**Keywords:** *Plasmodium falciparum*, *Anopheles stephensi*, Abscisic acid, TAK1, Nitric oxide, Lifespan, Transmission, Malaria, Innate immunity

## Abstract

**Background:**

Abscisic acid (ABA) is naturally present in mammalian blood and circulating levels can be increased by oral supplementation. We showed previously that oral ABA supplementation in a mouse model of *Plasmodium yoelii* 17XNL infection reduced parasitemia and gametocytemia, spleen and liver pathology, and parasite transmission to the mosquito *Anopheles stephensi* fed on these mice. Treatment of cultured *Plasmodium falciparum* with ABA at levels detected in our model had no effects on asexual growth or gametocyte formation in vitro. However, ABA treatment of cultured *P. falciparum* immediately prior to mosquito feeding significantly reduced oocyst development in *A. stephensi via* ABA-dependent synthesis of nitric oxide (NO) in the mosquito midgut.

**Results:**

Here we describe the mechanisms of effects of ABA on mosquito physiology, which are dependent on phosphorylation of TGF-β-activated kinase 1 (TAK1) and associated with changes in homeostatic gene expression and activity of kinases that are central to metabolic regulation in the midgut epithelium. Collectively, the timing of these effects suggests a transient physiological shift that enhances NF-κB-dependent innate immunity without significantly altering mosquito lifespan or fecundity.

**Conclusions:**

ABA is a highly conserved regulator of immune and metabolic homeostasis within the malaria vector *A. stephensi* with potential as a transmission-blocking supplemental treatment.

**Electronic supplementary material:**

The online version of this article (doi:10.1186/s13071-017-2276-4) contains supplementary material, which is available to authorized users.

## Background

We showed previously that oral supplementation of mice with the isoprenoid abscisic acid (ABA) increased circulating levels of ABA and reduced parasitemia, gametocytemia, and disease pathology as well as transmission of *Plasmodium yoelii* 17XNL to *Anopheles stephensi* [1]. In parallel studies, ABA had no direct effects on growth of cultured *P. falciparum* asexual stages or gametocyte development in vitro. In *A. stephensi*, ABA ingestion modestly induced expression of two antimicrobial peptide genes and markedly (13-fold) induced *nitric oxide synthase* (*nos*) midgut transcript levels in mosquitoes fed on supplemented *P. falciparum* culture relative to controls at 4–6 h post-feeding. Addition of the NOS inhibitor L-NAME confirmed that ABA-dependent NO synthesis in the mosquito midgut eliminated parasites prior to oocyst formation [1]. Given that *A. stephensi* NO synthesis is regulated by multiple signaling pathways with broad effects on mosquito physiology [2–4], the timing and mechanism(s) whereby ABA induces NO synthesis could provide significant insights into the potential effects of ABA on other life history traits that are important to vectorial capacity, including lifespan and egg production [5].

ABA is an ancient signaling molecule, with known biology in plants, invertebrates, and mammals, likely acting through pathways that have been conserved over evolutionary time. In mammalian studies, ABA has been shown to stimulate insulin release from pancreatic beta cells [6]. In *A. stephensi*, the insulin- and insulin-like growth factor (IIS) pathway can coordinately regulate anti-parasite defenses as well as mosquito lifespan and reproduction [7–13]. These effects are due, in part, to IIS-dependent changes in midgut intermediary metabolism, mitochondrial function, and epithelial homeostasis [10, 11, 13, 14]. ABA signaling in plants has been associated with activation of mitogen-activated protein kinases (MAPKs), which regulate pathogen sensing and developmental processes as well as senescence [15, 16]. Notably, nearly 25% of the 1500 ABA-regulated genes in plants derive from ABA-dependent activation of a single MAP2K known as MKK3 [15]. ABA signaling also impacts intermediary metabolism in plants, with increasing evidence for anterograde and retrograde signaling as well as changes in mitochondrial function that can impact plant defenses [17–19]. In *A. stephensi*, p38 MAPK and extracellular signal-regulated kinase (ERK MAPK) are involved in pathogen sensing and NOS activation [20, 21]. Both ERK and p38 MAPK are known to be regulated by transforming growth factor-β-associated kinase 1 (TAK1), a MAP3K that also regulates NOS activation in *A. stephensi* [22–25]. Given that IIS and MAPK signaling control metabolism and mitochondrial function in *A. stephensi*, ABA signaling through these pathways could coordinately regulate defense, lifespan, and reproduction in the mosquito host.

Lifespan is a determinant of the total number of eggs laid by a female mosquito and the probability that she will survive the extrinsic incubation period, or the time required for development of infectious sporozoites, following a blood meal. Changes in mosquito lifespan have a fourth order effect on vectorial capacity, with senescence notably increasing this effect size [5, 26, 27]. Reduced egg production yields fewer adults in the next generation, which impacts biting rate and, therefore, parasite transmission. Accordingly, we sought to characterize ABA signaling in *A. stephensi* and the life history traits that are connected by these cellular pathways to better understand the effects of ABA on this important vector species.

## Methods

### Reagents and chemicals

Anti-phospho-AMPKα Thr172 (#2535), phospho-TAK1 Thr184 (#4537), phospho-GSK-3α/β Ser21/9 (#9331), and phospho-Akt Ser473 (#9271) antibodies were purchased from Cell Signaling Technology (Danvers, MA, USA). Anti-phospho-p70S6K Thr412 (#07–018) and phospho-FOXO Thr32 (#07–694) were purchased from Millipore (Billerica, MA, USA). Anti-phospho-ERK (#M8159) was purchased from Sigma-Aldrich (St. Louis, MO, USA). Anti-phospho-JNK (#44-682G) was purchased from Biosource (Carlsbad, CA, USA). Anti-phospho-p38 MAPK Thr180/Tyr182 (#10009177) was purchased from Cayman Chemical. Anti-GAPDH (#ab36840) was purchased from Abcam (Cambridge, MA, USA). Goat anti-rabbit secondary antibody (#ALI4404) was purchased from Biosource. Rabbit anti-mouse secondary antibody (#A9044) was purchased from Sigma-Aldrich. Morpholinos were purchased from Gene Tools, LLC (Philomath, OR, USA).

### *Plasmodium falciparum* infections

Three- to five-day-old *A. stephensi* were provided with *P. falciparum*-infected blood containing 100 nM ABA or a diluent control as previously described [1]. Non-engorged individuals were removed immediately after feeding. Midguts were dissected at various time points for gene expression and protein analysis. Oocysts were counted on dissected midguts stained with 0.5% mercurochrome at 10 days post-feeding.

For TAK1-knockdown experiments mosquitoes were fed a saline-ATP meal with 10 μM *A. stephensi* TAK1-targeted (5′-GAT CCT TAT TAC GTT TCG CTT CGT A-3′) or control human beta-globin-targeted (5′-CCT CTT ACC TCA GTT ACA ATT TAT A-3′) vivo morpholinos as previously described 3 days before being provided with a *P. falciparum*-infected blood meal [28].

### qRT-PCR assays

Transcript levels of *P. falciparum a18s* rDNA, *pfs16* and *pfs25* in infected mosquito midguts were determined by qRT-PCR [14]. Data were normalized to transcript levels for *A. stephensi* ribosomal s7 protein, *a18s* rDNA, and control levels as previously described [12]. *nos*, *defensin*, *apl1*, *tep1*, and *lrim* transcript levels were determined by qRT-PCR and analyzed as described previously [1].

### Western blotting assays

Western blots were prepared and analyzed as described [21]. Briefly, proteins were extracted from 10 pooled midguts, separated by gel electrophoresis on a 10% sodium dodecyl sulfate-polyacrylamide gel, transferred onto a nitrocellulose membrane (BioRad, Hercules, CA, USA) and blocked in 5% dry milk in Tris-buffered saline-0.1% Tween 20 (TBST) for 1 h at room temperature. Membranes were incubated overnight at 4 °C in primary (1°) and secondary (2°) antibodies diluted in 5% non-fat dry milk/TBST as follows: 1:1000 phospho-TAK1 and 1:10,000 goat anti-rabbit IgG; 1:1000 phospho-GSK-3 and 1;10,000 goat anti-rabbit IgG; 1:1000 phospho-p70S6K and 1:2000 goat anti-rabbit IgG; 1:1000 phospho-FOXO and 1:2000 goat anti-rabbit IgG; 1:1000 phospho-Akt and 1:5000 goat-anti-rabbit IgG; 1:10,000 phospho-ERK and 1:20,000 rabbit anti-mouse IgG; 1:1250 phospho-JNK and 1:20,000 goat anti-rabbit IgG; 1:1250 phospho-p38 MAPK and 1:20,000 goat-anti-rabbit IgG; 1:10,000 anti-GAPDH and 1:20,000 goat-anti-rabbit IgG. For detection of phospho-AMPK, membranes were incubated in 1:1000 goat anti-rabbit IgG in 5% milk (TBST) for 2 h at room temperature before imaging. All data were normalized to GAPDH levels in the same samples and to target protein levels in matched control midguts.

### Lifespan and fecundity measurements

Uninfected female *A. stephensi* were given the opportunity to bloodfeed once per week on a 1:1 vol: vol mixture of washed human red blood cells (RBCs) and phosphate-buffered saline (PBS) supplemented with 100 nM ABA or with an equivalent volume of diluent control. Mosquitoes were maintained on 10% sucrose solution and allowed to oviposit once per week. Dead individuals were removed and counted three times per week. For analyses of lifespan under nutrient stress, mosquitoes were treated as above but maintained on 3% sucrose solution between weekly blood meals.

To analyze the effects of ABA on infected lifespan, 3–5 day old mosquitoes were provided with a *P. falciparum*-infected blood meal supplemented with 100 nM ABA or with an equivalent volume of diluent control. Mosquitoes that did not fully engorge were removed from the cartons. For the remainder of the infected lifespan study, mosquitoes were given the opportunity to feed once per week on uninfected blood with or without ABA. Mosquitoes were allowed to oviposit once per week and maintained on 10% sucrose solution between weekly blood meals. Dead individuals were counted every 24 h and each dead individual was placed in Trizol for subsequent RNA isolation. Infection status of each mosquito was determined by detection of transcript levels of *P. falciparum* mitochondrial cytochrome *c* oxidase subunit 1 (*Pfcox1*) gene (forward: 5′-TGC CAG GAT TAT TCG GAG GA-3′; reverse: 5′-CCA TCC AGT TCC ACC ACC AA-3′) by qRT-PCR [29].

For fecundity studies, 3–5 day old female *A. stephensi* were fed on uninfected RBCs in PBS (1:1 vol: vol) supplemented with or without 100 nM ABA. Fully engorged females were housed individually and provided oviposition cups at 2 days post-feeding. Two days after the provision of oviposition cups, eggs were counted and prepared for hatching in individual water cups. First instar larvae were provided with a 2% solution of 2:1 Sera Micron® powdered fish food (Sera North America, Montgomeryville, PA, USA) and baker’s yeast. First instar larvae were counted at 2 days after hatching.

### Statistical analyses

Levels of phosphorylated proteins were analyzed by Student’s t-test. *Nos* transcript levels were analyzed by Wilcoxon matched-pairs signed rank test. All other qRT-PCR data were analyzed by Student’s t-test. Infection prevalence, egg laying rate, and egg hatch rate were analyzed using Fisher’s exact test. Median survival rate and clutch size were analyzed by Wilcoxon matched-pairs signed rank test and Student’s t-test, respectively. Lifespan data were analyzed using log rank test and Gehan-Breslow-Wilcoxon test. Differences were considered significant at *P* < 0.05.

## Results

### Sexual stage commitment of *P. falciparum* is restricted by ABA treatment within 24 h of mosquito infection

**Fig. 1:**
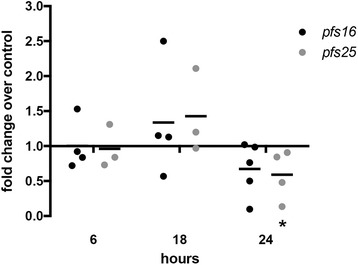
Sexual stage commitment of *P. falciparum* is restricted by ABA treatment within 24 h of mosquito infection. Expression levels of *P. falciparum* genes *pfs16* and *pfs25* in ABA-supplemented parasite-fed mosquito midguts were normalized to mosquito *ribosomal s7 protein* gene and parasite *a18s* gene expression. Each dot represents one replicate of 10 pooled midguts. Data are shown as fold change relative to control and were analyzed by Student’s t-test. *P ≤ 0.05

The *P. falciparum* genes *pfs16* and *pfs25* are expressed in early gametocytes through zygotes and in gametocytes through oocyst stages, respectively [30, 31]. In ABA-treated mosquitoes, expression levels of *pfs16* trended downward and *pfs25* transcript levels were significantly lower than controls by 24 h post-infection (*t* = 2.311, *df* = 3, *P* = 0.05), suggesting that ABA decreased parasite numbers between 18 and 24 h post-infection, prior to parasite invasion of the midgut epithelium (Fig. 1) [32].

### ABA reduced *Plasmodium* infection in the mosquito by increasing TAK1-regulated host immunity

**Fig. 2:**
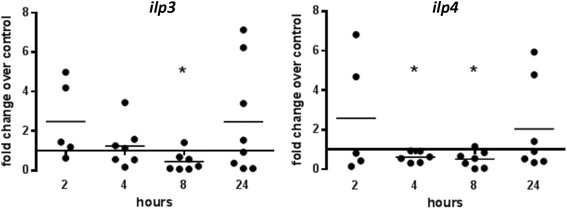
ABA reduced insulin-like peptide gene expression in the midgut. *ilp3* and *ilp4* mRNA expression levels in ABA-treated, *P. falciparum-*fed mosquito midguts are shown as fold changes compared to control-treated mosquitoes. Each dot represents one replicate of 10 pooled midguts. Data were analyzed by Student’s t-test. *P < 0.05

**Fig. 3:**
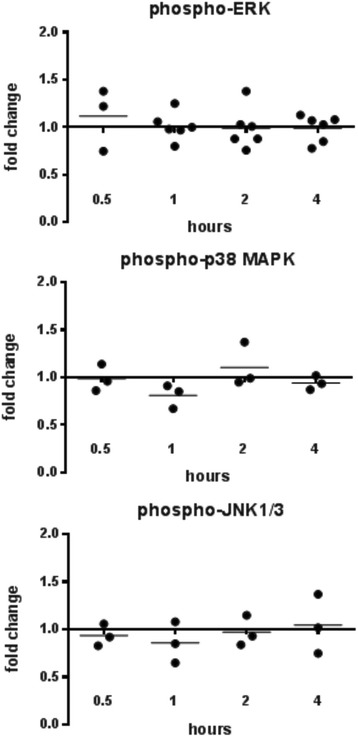
ABA did not alter MAPK phosphorylation in the midgut. Levels of phosphorylated ERK, JNK, and p38 MAPK in midguts of ABA-supplemented *P. falciparum*-fed mosquitoes are shown as fold-changes compared to controls. Each dot represents one replicate of 15 pooled midguts. Data were analyzed by Student’s t-test

**Fig. 4:**
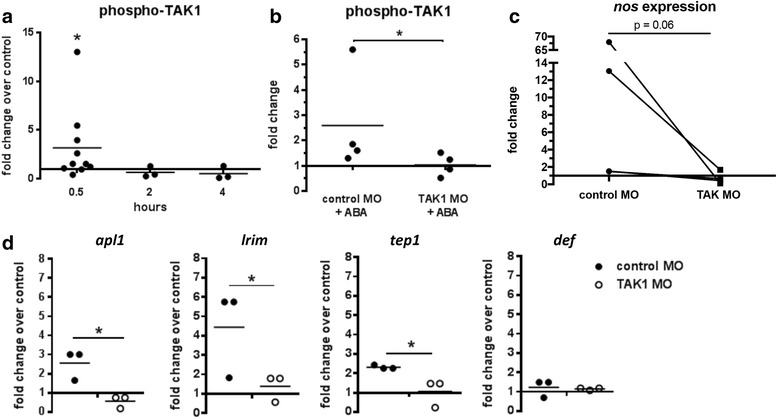
ABA increased TAK1 phosphorylation upstream of NF-κB-dependent gene expression. **a** Levels of TAK1 phosphorylation in ABA-supplemented parasite-fed mosquito midguts are shown as fold changes compared to controls. Each dot represents one replicate of 15 pooled midguts. **b** Fold changes in levels of TAK1 phosphorylation in the midguts of ABA-supplemented, parasite-fed mosquitoes at 30 min post-infection, normalized to non-supplemented, parasite-fed controls, treated with a control or TAK1-targeted morpholino (MO). **c** Fold changes in expression of *nos* mRNA in ABA-supplemented parasite-fed mosquito midguts (6 h post-infection) fed either control- or TAK1-morpholinos (*n* = 4). **d** Fold change in expression levels of *apl1*, *lrim*, *tep1*, and *defensin* in ABA-supplemented *P. falciparum*-fed mosquitoes normalized to non-supplemented, parasite fed controls, fed either a control- or TAK1-morpholino. Each dot represents one replicate of 10 pooled midguts. Data were normalized to their respective morpholino controls. Phosphorylated TAK1 levels were analyzed by Student’s t-test. *nos* expression was analyzed by Wilcoxon matched-pairs signed rank test. *apl1*, *lrim*, *tep1* and *defensin* levels were analyzed by Student’s t-test. *P < 0.05

We previously showed that ABA supplementation increased expression of *defensin, apl1,* and *nos* in the midgut of *A. stephensi* within hours of blood-feeding*.* These genes are regulated by multiple pathways, including those that are IIS-, MAPK- and NF-κB-dependent [12–14, 20, 21]. To determine whether ABA activates these pathways to regulate host immunity, we examined *insulin-like peptide* (*ilp*) gene expression, which is regulated by IIS [12], as well as activation of the MAPKs ERK, JNK, and p38 MAPK and the MAP3K TAK1 in the *A. stephensi* midgut. We focused our analyses on ILP3 and ILP4, which regulate *P. falciparum* development in *A. stephensi* through effects on midgut intermediary metabolism and mitochondrial function [13]. In contrast to ABA enhancement of insulin secretion in mammalian cells, ABA significantly repressed expression of *ilp3* mRNA at 8 h post-infection (*t* = 2.953, *df* = 6, *P* = 0.026) and *ilp4* mRNA at 4 (*t* = 3.472, *df* = 5, *P* = 0.018), 6 (*t* = 13.42, *df* = 5, *P* < 0.0001), and 8 (*t* = 3.079, *df* = 6, *P* = 0.022) hours post-infection in the mosquito midgut relative to controls (Fig. 2). This repression was consistent with ABA’s effect on infection given that knockdown of ILP3 and ILP4 is also associated with reduced *P. falciparum* infection in *A. stephensi* [12]. Addition of 100 nM ABA to a *P. falciparum*-infected blood meal had no effect on phosphorylation of ERK, JNK or p38 MAPK relative to controls (Fig. 3, Additional file 1: Figure S1), but ABA treatment rapidly increased phosphorylation of TAK1 with a significant 3-fold mean increase occurring within 30 min post-infection (*t* = 1.804, *df* = 9, *P* = 0.05) (Fig. 4a, Additional file 1: Figure S1).

To determine whether TAK1 phosphorylation mediated ABA-dependent immune gene expression and the effects of ABA on *P. falciparum* infection, we used a knockdown strategy based on supplementation of an infected blood meal with TAK1-targeted morpholino. Although phosphorylated TAK1 is expected to constitute a relatively small proportion of total TAK1 protein, TAK1 morpholino-treated, infected *A. stephensi* had significantly reduced midgut phospho-TAK1 levels in response to ABA relative to infected mosquitoes treated with control morpholino (Mann-Whitney test, *U* = 1, *n*_1_ = 4, *n*_2_ = 4, *P* = 0.029) (Fig. 4b), confirming TAK1 knockdown. In TAK1 morpholino-treated, infected mosquitoes, ABA-dependent *nos* expression trended downward (Wilcoxon test, *W* = -10, *P* = 0.06) (Fig. 4c) and expression levels of *apl1* (*t* = 4.074, *df* = 4, *P* = 0.008), *lrim* (*t* = 2.23, *df* = 4, *P* = 0.023), and *tep1* (*t* = 3.02, *df* = 4, *P* = 0.039) were significantly reduced relative to controls by 6–8 h post-infection (Fig. 4d). *defensin* expression, which was only modestly induced by ABA in earlier studies, was not altered by TAK1 knockdown (Fig. 4d).

**Fig. 5:**
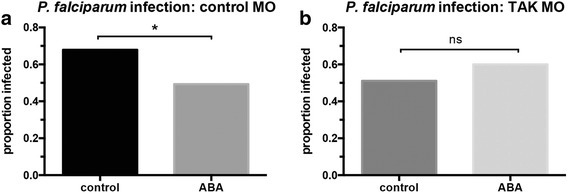
TAK1 knockdown prevented the ABA-dependent reduction of *P. falciparum* infection prevalence. The proportions of *P. falciparum-*infected mosquitoes fed **a** control morpholinos or **b** TAK1-targeted morpholinos with and without ABA supplementation in the infected bloodmeal were analyzed by Fisher’s exact test (*n* = 50–90 midguts). *P < 0.05

These observations suggested that ABA signaling of TAK1 phosphorylation controlled NF-κB-dependent immunity in *A. stephensi*. To link this signaling to *P. falciparum* infection, we examined infection prevalence (the presence of at least one oocyst) in TAK1 morpholino-treated and control mosquitoes with and without ABA supplementation. In control morpholino-treated mosquitoes, ABA supplementation as expected significantly reduced infection prevalence relative to mosquitoes not treated with ABA (Fisher’s exact test, *P* = 0.001) (Fig. 5a). In TAK1 morpholino-treated mosquitoes, however, ABA supplementation did not significantly alter infection prevalence compared to controls that were not treated with ABA (Fig. 5b). Taken together, these observations suggested that ABA-induced TAK1 phosphorylation mediates ABA repression of *P. falciparum* infection. Importantly, infection prevalences in both control morpholino-treated and TAK1 morpholino-treated mosquitoes in the absence of ABA were not significantly different from control mosquitoes that were not fed morpholinos (Additional file 2: Figure S2). In the absence of ABA treatment, infection levels in TAK1 morpholino-treated mosquitoes trended lower than those in mosquitoes treated with control morpholinos (Additional file 2: Figure S2). These observations suggested that reducing TAK1 may activate a compensatory immune response that is independent of the ABA-induced response.

### ABA transiently altered metabolism and homeostasis in the mosquito midgut

**Fig. 6:**
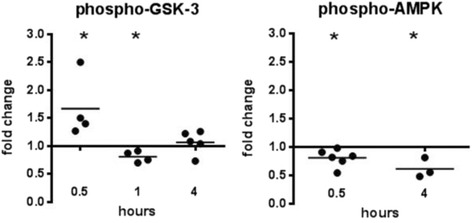
ABA altered phosphorylation of AMPK and GSK3 in the *A. stephensi* midgut. Fold changes in phosphorylated AMPK and GSK3 in the midguts of ABA-treated, *P. falciparum*-infected mosquitoes are shown compared to controls. Each dot represents one replicate of 15 pooled midguts. Data were analyzed by Student’s t-test. *P < 0.05

While ABA repression of *ilp3* and *ilp4* transcript levels in the *A. stephensi* midgut was consistent with the effects of ABA on *P. falciparum* infection, our previous studies of these peptides [13] as well as observed effects of ABA on mammals and plants [33–36] suggested that ABA may have broader effects on *A. stephensi* physiology. A major target of IIS is phosphorylation and inhibition of GSK-3, a pivotal mediator of metabolism, inflammation, cell death, and survival in mammalian cells [37]. Given that IIS is activated in the mosquito midgut within minutes by *P. falciparum* glycosylphosphatidylinositols (GPIs) [38], it was not surprising to see a trend towards increased inhibitory phosphorylation of GSK-3 in the midgut within 30 min of feeding on *P. falciparum*-infected blood with ABA (Fig. 6, Additional file 1: Figure S1). However, by 1 h post-feeding, GSK-3 inhibitory phosphorylation was significantly repressed in the midgut of ABA-treated mosquitoes relative to controls (*t* = 3.897, *df* = 3, *P* = 0.03), suggesting a shift to positive regulation of GSK-3 activity by ABA as has been observed in plants [39].

GSK-3 functions as a monitor of cellular energy status and under energy-rich conditions promotes dephosphorylation of AMP kinase (AMPK) at Thr172 [40], which inhibits AMPK activity. Inhibition of AMPK prevents the activation of catabolic pathways that generate ATP, the induction of mitochondrial biogenesis, and the inhibition of biosynthetic pathways that consume ATP. Accordingly, we examined AMPK in the midgut and found that Thr172 phosphorylation was significantly reduced in ABA-treated *A. stephensi* relative to controls at both 30 min (*t* = 3.145, *df* = 5, *P* = 0.0175) and 4 h post-feeding (*t* = 3.852, *df* = 2, *P* = 0.03) (Fig. 6, Additional file 1: Figure S1). Sustained inactivation or deficiency of AMPK promotes mitochondrial dysfunction, which can lead to elevated levels of mitochondrial reactive oxygen species (ROS) and inflammatory levels of NO [41, 42]. ABA-TAK1 signaling (Fig. 4) and AMPK inhibition (Fig. 6), therefore, could additively generate high levels of NO that, based on our previous observations [14], would be damaging to cell health and autophagic repair in the midgut epithelium.

**Fig. 7:**
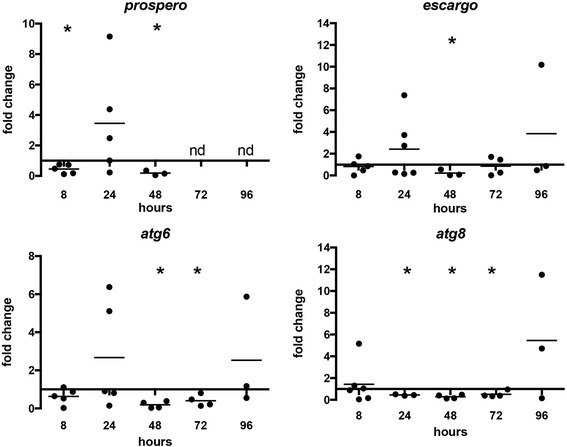
ABA reduced expression of genes involved in midgut epithelial cell replication, differentiation, and autophagy. mRNA levels of *prospero*, *escargot*, *atg6*, and *atg8* in ABA-treated *P. falciparum-*fed mosquito midguts are shown as fold changes compared to control mosquitoes. Each dot represents one replicate of 10 pooled midguts. Data were analyzed by Student’s t-test. *P < 0.05

Based on these suppositions, we examined readouts of midgut epithelial homeostasis [10, 11, 14] in *P. falciparum*-infected *A. stephensi* with and without ABA supplementation. We measured transcript levels of *prospero* and *escargot*, markers of cell proliferation and differentiation, respectively, as well as *atg6* and *atg8*, markers of autophagosome initiation and maturation, respectively [11, 14, 43–45]. Collectively, we have linked changes in thee markers to changes in ROS levels and midgut barrier integrity in the context of insulin-like growth factor treatment and Akt overexpression [11, 14]. In accordance with our observations of the transient effects on midgut signaling and gene expression (Figs. 2, 4), ABA significantly reduced expression of *prospero* (*t* = 9.26, *df* = 2, *P* = 0.0115) and *escargot* (*t* = 4.689, *df* = 2, *P* = 0.0426) at 48 h post-feeding and of *atg6* and *atg8* from 48 to 72 h (*t* = 9.181, *df* = 3, *P* = 0.0027; *t* = 3.938, *df* = 3, *P* = 0.0292) and 24–72 h post-feeding (*t* = 14.82, *df* = 2, *P* = 0.0045; *t* = 8.126, *df* = 3, *P* = 0.0039; *t* = 3.329, *df* = 3, *P* = 0.0447), respectively, with a return to baseline levels by 96 h post-feeding (Fig. 7).

### ABA does not alter mosquito lifespan or fecundity

**Fig. 8:**
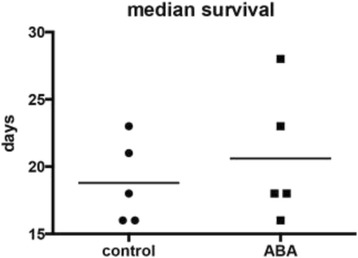
ABA supplementation did not alter mosquito median survival. Median survival (days) of control and ABA-treated mosquitoes from five lifespans (Table 1) are shown for mosquitoes that received weekly control or ABA-supplemented uninfected blood meals. Each lifespan was conducted with 300 female mosquitoes per treatment. Data were analyzed by Wilcoxon matched-pairs signed rank test

**Fig. 9:**
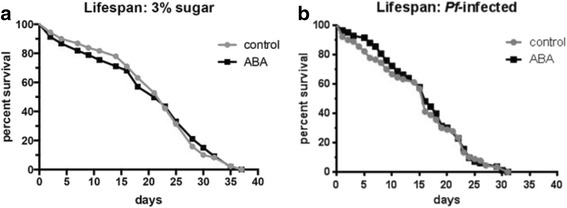
ABA supplementation did not alter *A. stephensi* lifespan in the context of nutrient stress or *Plasmodium falciparum* infection. **a** Survival curves are shown for mosquitoes provided weekly control or ABA-supplemented uninfected blood meals and maintained on 3% sucrose solution (*n* = 300 mosquitoes per control and ABA treatment groups). **b** Survival curves are shown for *P. falciparum*-infected mosquitoes provided control or ABA-supplemented weekly blood meals (*n* = 100 mosquitoes per control and ABA treatment groups). Infection status was confirmed by detection of *Pfcox1* in individual mosquitoes by qRT-PCR. Data were analyzed by log-rank test and Gehan-Breslow-Wilcoxon test

Although the effects of ABA on the midgut were transient, they were associated with a significant inflammatory response and notable effects on biomarkers of cellular homeostasis. Accordingly, we investigated the effect of ABA on mosquito lifespan and fecundity in a series of studies. Provision of 100 nM ABA in weekly, uninfected blood meals with maintenance between blood meals on 10% sucrose did not alter median mosquito survival although there was some variation among cohorts of mosquitoes (Fig. 8, Table 1, Additional file 3: Figure S3). Though ABA had no effect on lifespan under these conditions, we reasoned that ABA could alter lifespan when combined with nutrient stress (3% sugar) between blood meals [46, 47]. However, ABA had no effect on uninfected lifespan under nutrient stress (Fig. 9a).

**Table 1:** ABA supplementation did not alter *A. stephensi* lifespan. Median survival times of control and ABA-treated mosquitoes are shown from five lifespans in which mosquitoes received a weekly uninfected blood meal with or without ABA supplementation. Differences in lifespan between treatment groups were analyzed by log-rank test and Gehan-Breslow-Wilcoxon test

Replicate	Median survival (days)		*P*-value	
	ABA	Control	Log-rank test	Gehan-Breslow-Wilcoxon test
1	21	28	< 0.0001	< 0.0001
2	16	18	0.0012	0.0540
3	18	18	0.531	0.425
4	23	23	0.231	0.288
5	16	16	0.093	0.189

Extension of infected lifespan can increase parasite transmission, but the relationship between age-dependent mortality and infection is complex [48]. Accordingly, we conducted a lifespan study in which the first blood meal contained *P. falciparum*-infected RBCs with and without 100 nM ABA. Subsequent weekly blood meals were uninfected with and without ABA. As individual mosquitoes died they were collected and tested for parasite infection by detection of *Pfcox1* by qRT-PCR. Mosquitoes that did not have detectable levels of *Pfcox1* were considered uninfected and not included in the analysis. Even in the presence of parasite infection, however, ABA supplementation had no significant effect on the lifespan of *A. stephensi* (Fig. 9b).

**Fig. 10:**
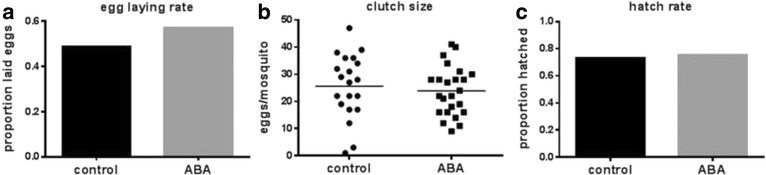
ABA supplementation did not alter *A. stephensi* egg production or viability. **a** Proportions of fully engorged mosquitoes (*n* = 41–42 females) are shown that laid one or more eggs within two days of feeding on control or ABA-supplemented uninfected blood. **b** Number of eggs laid after a single control or ABA-supplemented blood meal. Each dot represents the clutch size of a single mosquito. **c** Proportion of eggs that hatched to first instar larvae (*n* = 512–572). Egg laying rate and hatch rate were analyzed using Fisher’s exact test. Clutch size was analyzed by unpaired t-test

In addition to lifespan, mosquito population size can have a large impact on disease transmission. We reasoned that the changes in *ilp* expression induced by ABA could have an effect on fecundity as observed in *Aedes aegypti* [49] and based on observations that injections of ABA reduced vitellogenesis in the flesh fly *Sarcophaga bullata* [50]*.* However, ABA had no effect on egg laying rate, clutch size, and egg hatch rate relative to unsupplemented control *A. stephensi* following a single uninfected blood meal (Fig. 10).

## Discussion

**Fig. 11:**
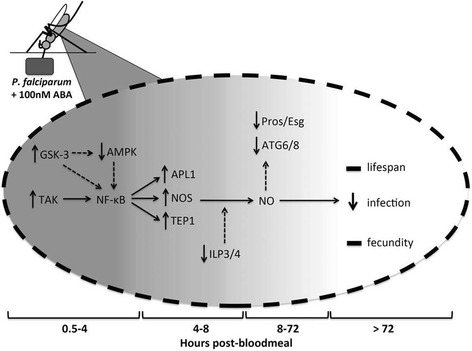
Model of ABA signaling within the mosquito midgut upon ingestion of a *P. falciparum*-infected bloodmeal. Within minutes of feeding, ABA increases TAK1 and GSK3 activity levels within the midgut. Active GSK3 reduces phosphorylation and activity of AMPK. TAK1 signaling increases NF-κB activity and downstream immune gene expression (*apl1*, *nos*, *tep1*), which is enhanced by the changes in GSK3 and AMPK levels. NOS catalyzes the synthesis of NO which kills parasites and transiently damages the midgut, as evidenced by decreased cellular replication and differentiation (*pros*/*esg*) and autophagy (*atg6/8*). However, these changes are temporally limited and ultimately have no effect on lifespan. Reduced levels of *ilp3* and *ilp4* are conducive to increased *nos* expression and reduced infection, but are short-lived and have no effect on fecundity

The effects of ABA ingestion on mosquito midgut signaling and gene expression are both notable and transient (Fig. 11). An early transient response to ABA is also seen in plants where signaling through pathways, including the MAPK cascade, is induced within minutes of treatment and peaks within a few hours, followed by later transcriptional changes that control stress responses by maintaining redox and water balance [51]. Factors that terminate ABA signaling in plants are poorly understood, although target-specific protein degradation appears to contribute to downregulation of induced stress responses [52]. Although it is unclear whether inhibitory signaling cascades feedback to inhibit ABA signaling in *A. stephensi* or if these short-lived responses reflect limited retention of ABA or degradation of ABA or induced target proteins, the biological effects of ABA on the mosquito are nonetheless significant.

Within 30 min to 1 h post-feeding, ABA induced changes in phosphorylation and activity of major immune and metabolic regulators in the mosquito midgut, including TAK1, AMPK, and GSK-3. ABA supplementation increased levels of phosphorylated TAK1, a positive regulator of NF-κB signaling, and TAK1 knockdown in the mosquito midgut abrogated the effects of ABA on immune gene expression and *P. falciparum* infection prevalence. These data support the hypothesis that ABA enhances anti-parasite defenses by increasing TAK1-dependent expression of NF-κB-regulated immune genes, including *nos*. These data are also consistent with findings in mammalian cells, where ABA treatment can enhance NF-κB nuclear translocation and transcriptional activity in multiple cell types [53–55].

The changes in metabolic protein activity and gene expression in response to ABA suggest a temporary shift in bioenergetic regulation within the midgut that facilitates and perhaps enhances parasite clearance. Specifically, a reduction in AMPK phosphorylation within 30 min post-ingestion of ABA can promote mitochondrial NO production [41, 42], an effective anti-parasite defense in *A. stephensi* [14]. Further, ABA-dependent activation of GSK3 by 1 h post-feeding would be predicted to sustain dephosphorylation of AMPK, enhancing NF-κB activity and NO production through alternate signaling pathways [40, 56–61]. Combined, these predicted synergistic effects on NO synthesis would support effective killing of *P. falciparum* sexual stages prior to invasion of the midgut epithelium. The repression of AMPK activity is also consistent with the repression of autophagy [62] observed 24–72 h post-feeding (Fig. 7). Interestingly, inhibition of autophagy can increase caspase-dependent apoptotic cell death [63, 64]. Together with high levels of NO synthesis, an increase in apoptosis in the midgut epithelium during ookinete invasion would be predicted to additively reduce oocyst formation in the *A. stephensi* midgut [65].

We have observed that inflammatory NO synthesis can also damage the *A. stephensi* midgut epithelium, with concomitant reductions in stem cell proliferation and differentiation (*escargot*, *prospero*). Increased NF-κB activity has also been linked with decreased expression of autophagy-related genes as well as decreased cell renewal and differentiation [66, 67], suggesting multiple mechanisms whereby ABA could alter midgut homeostasis. In *A. stephensi* engineered to overexpress Akt under a midgut-specific promoter [68], damage to midgut mitochondrial function and epithelial homeostasis was profound and sustained, resulting in a significant reduction in lifespan. Here we saw no effect of ingested ABA on lifespan, even under the stresses of nutrient limitation and *P. falciparum* infection, most likely because the effects of ABA on signaling and gene expression were transient. Similarly, ABA had no effect on egg laying or egg viability within the first gonotrophic cycle, providing further evidence that transient enhancement of host defenses by ABA does not significantly alter fitness. Importantly, since lifespan and reproduction in the first gonotrophic are also not *increased*, the use of ABA as a therapeutic with transmission blocking activity would not be expected to increase vectorial capacity in mosquitoes that might feed on treated individuals.

Overall, our studies have demonstrated that the effects of ABA on mosquito immunity and key metabolic regulatory kinases are consistent with effects that have been reported in plants and in mammals. Interestingly, in plants ABA can activate or inhibit AMPK signaling in a tissue dependent manner [69], suggesting that the effects of ABA in the mosquito could vary across tissues. Further, pro- and anti-inflammatory effects of ABA have been reported for acute infection and chronic diseases, respectively, in mammals [70] suggesting that the effects of ABA in mosquitoes could vary depending on the nature of the infecting pathogen or in response to changes in associated microbiota.

## Conclusions

ABA is a highly conserved regulator of immune and metabolic homeostasis within the malaria vector *A. stephensi* with potential as a transmission-blocking supplemental treatment. Further investigation of ABA signaling will help determine its potential efficacy as a transmission-blocking antimalarial therapeutic and increase our understanding of a highly conserved mediator of immune and metabolic homeostasis.

## Additional files

Additional file 1: Figure S1. Representative western blots of phospho-JNK (46 kDa), phospho-ERK (44 kDa), phospho-p38 MAPK (43 kDa), phospho-TAK1 (82 kDa), phospho-AMPK (62 kDa), phospho-GSK3 (46 kDa), and GAPDH (37 kDa) in midguts of mosquitoes at 30 min post-infection with *P. falciparum* with or without 100 nM ABA. (PDF 872 kb)

Additional file 2: Figure S2. *P. falciparum* infection prevalence in mosquitoes fed no morpholino, a control morpholino, or a TAK1-targeted morpholino. Data were analyzed by Fisher’s exact test (*n* = 50–90 midguts). (PDF 650 kb)

Additional file 3: Figure S3. Survival curves of five lifespan experiments, each conducted with a separate biological cohort of 300 mosquitoes per treatment. Mosquitoes were provided with weekly uninfected bloodmeals containing 100 nM ABA or a diluent control. (PDF 1065 kb)
