# Supplementary figures and images for: Abscisic acid induces a transient shift in signaling that enhances NF-κB-mediated parasite killing in the midgut of Anopheles stephensi without reducing lifespan or fecundity
Source: Parasit Vectors. 2017 Jul 13;10:333. doi: 10.1186/s13071-017-2276-4 (PMC5508651; doi:10.1186/s13071-017-2276-4)

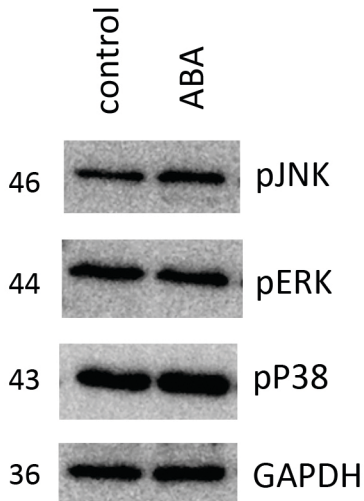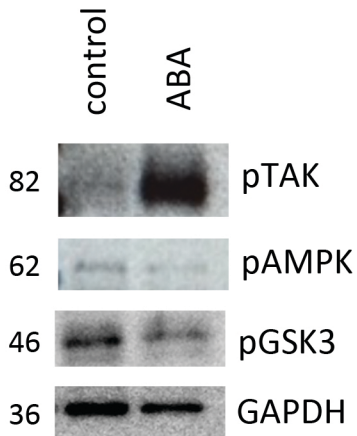

Supplement: Supplementary file 1 — Representative western blots of phospho-JNK (46 kDa), phospho-ERK (44 kDa), phospho-p38 MAPK (43 kDa), phospho-TAK1 (82 kDa), phospho-AMPK (62 kDa), phospho-GSK3 (46 kDa), and GAPDH (37 kDa) in midguts of mosquitoes at 30 min post-infection with P. falciparum with or without 100 nM ABA. (PDF 872 kb) [file 13071_2017_2276_MOESM1_ESM.pdf]

## *P. falciparum* infection

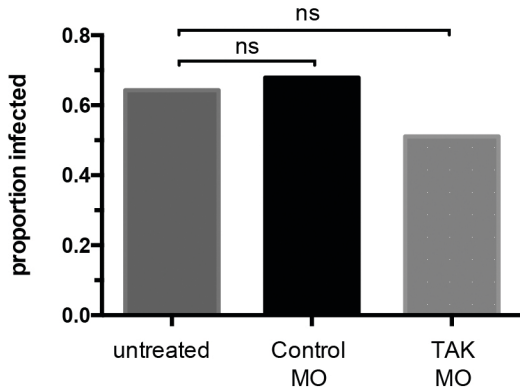

Supplement: Supplementary file 2 — P. falciparum infection prevalence in mosquitoes fed no morpholino, a control morpholino, or a TAK1-targeted morpholino. Data were analyzed by Fisher’s exact test (n = 50–90 midguts). (PDF 650 kb) [file 13071_2017_2276_MOESM2_ESM.pdf]

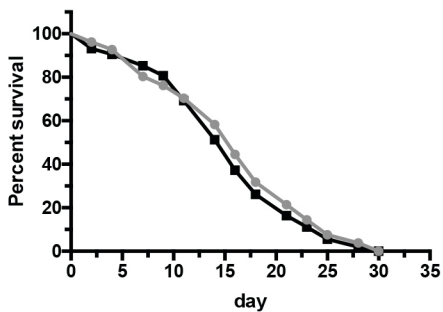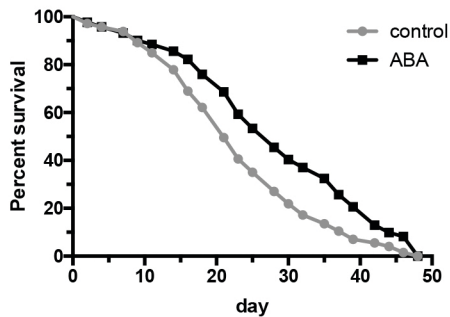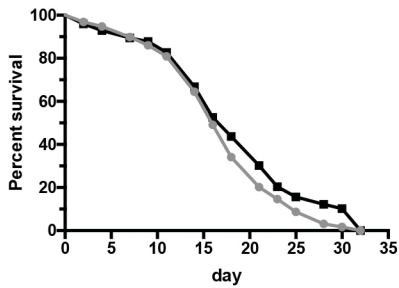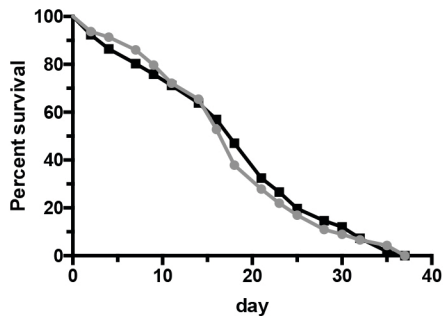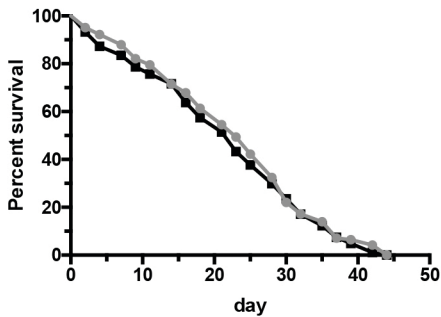

Supplement: Supplementary file 3 — Survival curves of five lifespan experiments, each conducted with a separate biological cohort of 300 mosquitoes per treatment. Mosquitoes were provided with weekly uninfected bloodmeals containing 100 nM ABA or a diluent control. (PDF 1065 kb) [file 13071_2017_2276_MOESM3_ESM.pdf]
